# Macrophage-secreted MMP9 induces mesenchymal transition in pancreatic cancer cells via PAR1 activation

**DOI:** 10.1007/s13402-020-00549-x

**Published:** 2020-08-18

**Authors:** Cansu Tekin, Hella L Aberson, Cynthia Waasdorp, Gerrit K J Hooijer, Onno J de Boer, Frederike Dijk, Maarten F Bijlsma, C Arnold Spek

**Affiliations:** 1 Center for Experimental and Molecular Medicine, Amsterdam UMC, University of Amsterdam, Amsterdam, The Netherlands; 2 Laboratory for Experimental Oncology and Radiobiology, Amsterdam UMC, University of Amsterdam, Cancer Center Amsterdam, Amsterdam, The Netherlands; 3 Oncode Institute, Amsterdam, The Netherlands; 4 Department of Pathology, Amsterdam UMC, University of Amsterdam, Amsterdam, The Netherlands

**Keywords:** Pancreatic cancer, PDAC, PAR1, MMP9, Macrophages, EMT

## Abstract

**Purpose:**

Targeting tumor-infiltrating macrophages limits progression and improves chemotherapeutic responses in pancreatic ductal adenocarcinoma (PDAC). Protease-activated receptor (PAR)1 drives monocyte/macrophage recruitment, and stromal ablation of PAR1 limits cancer growth and enhances gemcitabine sensitivity in experimental PDAC. However, the functional interplay between PAR1, macrophages and tumor cells remains unexplored. Here we address the PAR1-macrophage-tumor cell crosstalk and assess its contributions to tumor progression.

**Methods:**

PAR1 expression and macrophage infiltration were correlated in primary PDAC biopsies using gene expression datasets and tissue microarrays. Medium transfer experiments were used to evaluate the functional consequences of macrophage-tumor cell crosstalk and to assess the contribution of PAR1 to the observed responses. PAR1 cleavage assays were used to identify a macrophage-secreted PAR1 agonist, and the effects of candidate proteases were assessed in medium transfer experiments with specific inhibitors and/or recombinant agonist.

**Results:**

PAR1 expression correlates with macrophage infiltration in primary PDACs, and macrophages induce mesenchymal transition of PDAC cells through PAR1 activation. Protease profiling identified macrophage-secreted matrix metalloprotease 9 (MMP9) as the relevant PAR1 agonist in PDAC. PAR1 and/or MMP9 inhibition limited macrophage-driven mesenchymal transition. Likewise, preventing mesenchymal transition by silencing ZEB1 or by pharmacological inhibition of the MMP9/PAR1 axis significantly reduced the ability of tumor cells to survive the anti-tumor activities of macrophages.

**Conclusion:**

Macrophages secrete MMP9, which acts upon PDAC cell PAR1 to induce mesenchymal transition. This macrophage-induced mesenchymal transition supports the tumor-promoting role of macrophage influx, explaining the dichotomous contributions of these immune cells to tumor growth.

**Electronic supplementary material:**

The online version of this article (10.1007/s13402-020-00549-x) contains supplementary material, which is available to authorized users.

## Introduction

Pancreatic ductal adenocarcinoma (PDAC) is one of the most lethal cancers with reported 5-year survival rates of less than 8% [1, 2]. The high mortality rate of PDAC is mostly due to a late diagnosis with the vast majority of patients presenting with locally advanced or metastatic disease, and only around 20% of the patients are eligible for surgical resection of the tumor. Despite intense research efforts to improve and develop therapies, progress in therapeutic outcome has been slow, and current treatment options are still inadequate [2]. In the recent past, gemcitabine was used as first-line therapy, but survival benefits were limited [3]. Novel combination therapies like, for instance, FOLFIRINOX [4] or gemcitabine with Nab-paclitaxel [5], are superior over single-drug gemcitabine regimens, but even these intense combination treatments have shown limited efficacy. To increase PDAC survival rates, a better understanding of the mechanisms that contribute to the poor prognosis is urgently needed.

Macrophages are specialized mononuclear phagocytic immune cells critically involved in host defense and tissue homeostasis [6]. In cancer, macrophages have traditionally been considered to harbor cytotoxic activity, and numerous studies indeed show that macrophages may kill tumor cells by secreting cytotoxic molecules, such as TNF-α, IL-12, nitric oxide (NO), and reactive oxygen species (ROS) [7, 8]. Despite their cytotoxic capacity, however, macrophages are now increasingly believed to be pro-tumorigenic and to potentiate tumor growth [9–11]. High densities of macrophages are commonly seen in many different cancer types, including PDAC, and they are typically associated with poor prognosis [9]. Indeed, tumor-associated macrophages have been reported to induce epithelial-to-mesenchymal transition (EMT) [12–14], and blocking macrophage infiltration to decrease the number of metastatic lesions [15]. Furthermore, macrophage numbers have been reported to be associated with therapy resistance in pancreatic cancer [16, 17].

Protease-activated receptor (PAR)1, a seven-transmembrane G protein-coupled receptor (GPCR), is expressed in many tumor types, and its expression is associated with tumor progression and poor prognosis [18–21]. In contrast to most GPCRs, PAR1 activation requires proteolytic cleavage rather than classical ligand binding. PAR1 was initially identified as the thrombin receptor (F2R), but recently other agonists, such as activated protein C, matrix metalloproteases (MMPs), and kallikreins, have been described [22]. Agonist-dependent proteolytic removal of the N-terminal extracellular region of PAR1 releases a tethered ligand that interacts with the body of the receptor to activate signaling pathways that affect numerous pathophysiological responses. In PDAC, PAR1 is abundantly expressed in both primary tumors as well as in metastases, and genetic ablation of PAR1 from the tumor microenvironment limits cancer growth and enhances gemcitabine sensitivity in experimental animals [23]. Of note, stromal PAR1 deficiency is accompanied by a reduced macrophage infiltration into the tumor microenvironment, which is a direct effect of PAR1-dependent chemokine production [23]. In the current study, we explore the functional interplay between PAR1, macrophages and tumor cells. In doing so, we uncovered a mechanism through which macrophage-induced mesenchymal transition allows tumor cells to escape macrophage-dependent cell death.

## Materials and methods

### Patients

A tissue microarray was prepared from tumor specimens of pancreatic cancer patients obtained during surgery according to the guidelines of the Medical Ethical Committee of the Amsterdam University Medical Centers (Amsterdam UMC). We used anonymized formalin-fixed paraffin-embedded (FFPE) human tissue samples that were obtained during standard diagnostic procedures, and which were later made available for scientific research (so-called ‘further use’ of human tissue). According to the Code of Conduct for dealing with responsibility for human tissue in the context of health research, these biological materials are not subject to any requirement for ethical review or consent from patients [24]. From selected FFPE blocks with primary PDAC (*n* = 30), one core of tumor with a diameter of 1 mm was collected using a TMA instrument (Beecher Instruments, Silver Springs MD, USA) and inserted in a recipient block. Each recipient block was sectioned at 4 μm, and dried overnight at 37 °C.

### Immunohistochemistry

PDAC FFPE tissue sections were stained for PAR1 (ATAP-2: sc-13,503, Santa Cruz Biotechnology, Dallas, TX, USA), CD68 (PG-M1, Dako Omnis, Agilent, Santa Clara, CA) and CD163 (10D6, Thermo Fischer Scientific, Waltham, MA). Slides were deparaffinized and rehydrated, after which endogenous peroxidase activity was quenched in 0.5% hydrogen peroxide-methanol solution. Antigen retrieval was performed for 20 min in Tris/EDTA pH 9.0 buffer in a pressure cooker. Primary antibodies directed against CD163 and CD68 were applied for 60 min at room temperature, whereas an anti-PAR1 antibody was applied overnight at 4 °C. Slides were subsequently incubated with the appropriate HRP-conjugated secondary antibodies. To obtain triple staining of single slides, the same slide was sequentially stained. Following each staining, the sections were digitized (scanned) using a Philips IntelliSite UFS (Philips Digital Pathology Solutions, Best, the Netherlands) followed by a stripping step in which the colors and bound complexes were eluted from the sections, as described before [25]. De-stained sections were examined prior to the subsequent immunostaining to ensure that the previous staining was no longer present. From the acquired digital images, areas of interest were selected and aligned by non-linear registration of the separate images. After creating image stacks and color deconvolution for NovaRed, false-color images were created for each staining and re-stacked using Fiji/Image J analysis software (NIH, Bethesda, MD, USA).

### Reagents

The following reagents were used: GM6001 (ChemCruz, Dallas, TX, USA), Vorapaxar (250 nM, Adooq Bioscience, Irvine, CA, USA), L-glutamine (Lonza, Basel, Switzerland), Penicillin and Streptomycin (Lonza, Basel, Switzerland), Trypsin-EDTA (Gibco, Thermo Fischer Scientific, Waltham, MA, USA), Puromycin (Sigma, St. Louis, MO, USA), phorbol 12-myristate 13-acetate (PMA; Merck-Millipore, Burlington, MA, USA), 3-(4,5-dimethylthiazol- 2-yl)-2,5-diphenyltetrazolium (MTT), pre-activated recombinant MMP9 and Crystal Violet (both from Sigma, St. Louis, MO, USA).

### Cell culture

Human PANC-1, MIA PaCa-2 and Capan-2 pancreatic cancer cells (ATCC, Manassas, VA, USA) were cultured in high glucose (4.5 g/ml) DMEM (Gibco, Thermo Fischer Scientific, Waltham, MA, USA) and human THP-1 (ATCC, Manassas, VA, USA) cells were cultured in RPMI-1640 medium (Gibco, Thermo Fischer Scientific, Waltham, MA, USA). All media were supplemented with 10% fetal bovine serum (FBS, #F7524, Sigma, St. Louis, MO, USA) and L-glutamine (2 mM), except RPMI-1640 for conditioned media experiments, which was supplemented with 1x GlutaMAX (Gibco, Thermo Fischer Scientific, Waltham, MA, USA), penicillin (100 units/ml), and streptomycin (500 μg/ml) (Lonza, Basel, Switzerland) according to routine cell culture procedures. Cells were incubated in 5% CO_2_ incubators at 37 °C. All PDAC cell lines were authenticated by STR profiling (Promega PowerPlex, Leiden, Netherlands), and tested for mycoplasma by PCR monthly.

### Generation of macrophages and conditioned media

THP-1 cells seeded at 10^6^ in a T75 culture flask (Greiner Bio-One, Kremsmünster, Austria) were treated with 150 nM phorbol 12-myristate 13-acetate (PMA) for 24 h in RPMI-1640 (Gibco, Thermo Fischer Scientific, Waltham, MA, USA) medium. Next, adherent activated THP-1 cells were washed with fresh medium to remove PMA, and cells were cultured in fresh medium for another 24 h, after which the medium was refreshed once more. Conditioned medium from these M0 macrophages (M0-CM), which express CD68 and CD163 but not CD206 (Sup. Figure 2A), was collected 48 h later. After collection, M0-CM was centrifuged at 1200 rpm for 4 min to remove cell debris, filtered using 0.2 μm syringe filters (Corning, New York, NY, USA), and stored at 4 °C. For experimental procedures, the M0-CM medium was diluted 1:1 with fresh media to refresh the nutrients and serum content of the medium. M2 macrophages for phenotype testing (Sup. Figure 2A), were generated from THP-1 cells, as described previously [26].

### MMP9 cleavage prediction of PAR1

The FASTA sequence of human PAR1 (*F2R*), Uniprot ID: P25116, was loaded into the CleavePredict MMP substrate cleavage prediction tool for MMPs (http://cleavpredict.sanfordburnham.org [27]). After analysis, position weight matrices (PWM) representative of prediction scores for PAR1 cleavage were exported. In this analysis, higher PWM scores indicate a more substantial likelihood of cleavage at a specific site. A model representing the PAR1 N-terminal arm between amino acids 33 and 44, as shown in Fig. 4, was generated in SWISS-MODEL (https://swissmodel.expasy.org/) and agonist-specific cleavage sites were colored using PyMol (https://pymol.org).

### MMP9 ELISA

MMP9 concentrations in M0-CM were determined using a commercially available MMP9 ELISA kit (DY911–05, R&D Systems, Minneapolis, MN, USA) according to the manufacturer’s instructions.

### Quantitative real-time PCR

Total RNA was isolated using a NucleoSpin RNA miniprep kit (Macherey Nagel, Düren, Germany). cDNA was synthesized from DNase-treated total RNA using M-MLV-RT (Promega, Leiden, Netherlands) and random hexamers (Qiagen, Hilden, Germany). Real-time quantitative RT-PCR was performed using a Sensifast SYBR No-Rox Kit (Bioline, London, UK) on a LightCycler 480 II (Roche, Basel, Switzerland). Relative expression levels were calculated using the comparative threshold cycle (dCt method) and normalized for expression of the reference gene TBP. Primer sequences of the analyzed genes are shown in Supplementary Table 1.

### PDAC expression datasets

Gene expression datasets were derived from the Gene Expression Omnibus (http://www.ncbi.nlm.nih.gov/gds) using the R2 microarray analysis and visualization platform (http://r2.amc.nl). Pancreatic tumor expression datasets (GSE62452 [28], GSE28735 [29], GSE15471 [30], TCGA-PDAC [31], E-MTAB-6830 [32], GSE93326 [33] and GSE49149 [34]) were used for expression analysis of PAR1 (F2R), CD68 and CD163. The datasets were dichotomized for F2R, CD68, or CD163 based on the median expression and further analyzed on the same platform.

### Secreted alkaline phosphatase (SEAP) assay to detect PAR1 cleavage

HEK 293 cells stably expressing PAR1-SEAP (kindly provided by Dr. Mosnier, The Scripps Research Institute, La Jolla, CA, USA) were used for reporter assays as described previously [35]. PAR1-SEAP cells were incubated with 100 nM recombinant MMP9 (pre-activated, Sigma, St. Louis, MO, USA), 0.1 U/ml thrombin, or solvent control for 30 min in serum-free Opti-MEM (Gibco, Thermo Fischer Scientific, Waltham, MA, USA). Next, the supernatant was removed, and alkaline phosphatase activity was measured according to the manufacturer’s instructions using a Synergy HT Biotek Microplate Reader (Biotek Instruments, Winooski, VT, USA).

### Phase contrast microscopy and fluorescence microscopy

M0-CM-induced cellular changes were visualized at 20x magnification with phase-contrast on a Zeiss AxioVert microscope. Tracking cell behavior in time was performed with the scanning function on the EVOS® FL Cell Imaging System (Thermo Fischer Scientific, Waltham, MA, USA) at 10x magnification. Unlabelled Capan-2 cells were visualized with the phase contrast channel on the EVOS system. Scanned images were analyzed using ImageJ for area measurement (Capan-2).

### MTT cell proliferation and crystal violet cell viability assays

Cells at 70% confluence in 96-well plates were serum-starved overnight after which cell proliferation/viability was determined using 3-(4,5-dimethylthiazol- 2-yl)-2,5-diphenyltetrazolium (MTT; 5 mg/ml) or Crystal Violet (0.5% Crystal Violet in 6% Glutaraldehyde in PBS) at 72 h according to routine procedures. Cells were incubated with MTT reagent for 3 h or with Crystal Violet for 30 min. Measurements were performed using a Synergy HT Biotek Microplate Reader (Biotek Instruments, Winooski, VT, USA) at 560 nm for MTT and 600 nm for Crystal Violet. The decrease in proliferation/viability was calculated based on optical density at t = 0 as 0%.

### Flow cytometric detection of Annexin-V for apoptosis

After overnight serum starvation, cells were treated with M0-CM or control media for 48 h after which free-floating and attached cells were collected, re-suspended in Annexin-V binding buffer (BD, Franklin Lakes, NJ, USA) and transferred to 96-well plates for staining. To each well containing 100 μl cell suspension, 1 μl anti-Annexin-V FITC antibody (BD, Franklin Lakes, NJ) was added. After incubation in the dark for 1 h, cells were washed twice with Annexin-V binding buffer and re-suspended in 200 μl fresh buffer. Annexin-V positivity was next analyzed on a FACS Canto II (BD, Franklin Lakes, NJ, USA). The gating strategy for FITC positivity was set on the single-cell population (see Fig. 5C). The AnnexinV+ population was determined using antibody control samples (isotype control) applied to all conditions. For analysis, Geometric Mean Fluorescent Intensity (gMFI) on the FITC channel was used. Gating and data analysis were performed using FLOWJO v10 (FlowJo LLC, Ashland, OR, USA).

### Lentiviral gene silencing

PAR1 silenced PANC-1, MIA PaCa-2, and Capan-2 cells were established with knockdown efficiencies of around 70% for PANC-1 and Capan-2 and around 50% for MIA PaCa-2 cells [36]. For lentiviral silencing of *ZEB1,* shRNA clones TRCN0000017565 and TRCN0000017567 were used. Clone shc004 was used as control. Lentivirus was produced by transfecting HEK293T cells with 3rd generation transfer and packaging plasmids *pVSV*, *pMDL* and *pRES* using Lipofectamine 2000 (Thermo Fisher Scientific, Waltham, MA, USA). 48 and 72 h after transfection, the supernatant was harvested and 0.45 μm filtered (Millipore, Billerica, MA, USA). PANC-1 cells were transduced with 40 μl lentivirus and incubated for 48 h. Transduced cells were selected with 2 μg/ml puromycin (Sigma, St. Louis, MO, USA) for 72 h, after which the transduction efficiency was analyzed by qRT-PCR for the target gene.

### Statistical analysis

Data are presented as mean ± SEM. Statistical analysis was performed using GraphPad PRISM 8.0 (Graphpad Software Inc., La Jolla, CA, USA). Statistically significant differences were considered with a *p* value < 0.05. For further details of the statistical analyses, see figure legends. *P*-values are indicated by asterisks with * *p* < 0.05, ** *p* < 0.01, *** *p* < 0.001, and **** *p* < 0.0001.

## Results

### PAR1 expression correlates with macrophage markers in human pancreatic tumors

**Fig. 1:**
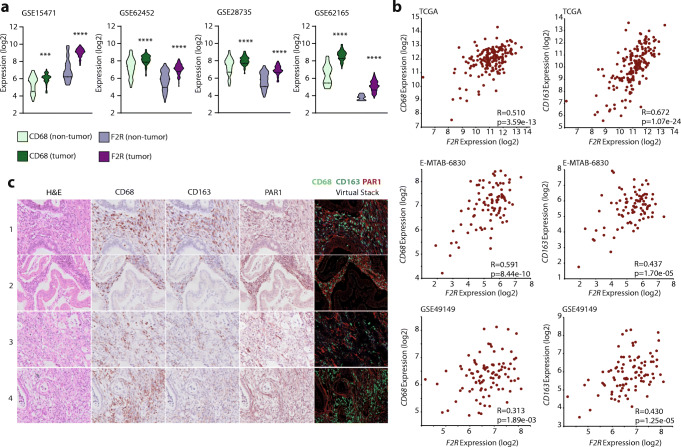
PAR1 is predominantly expressed in tumor tissue and correlates with macrophage markers. (**A**) Density plots for expression of CD68 (green) and PAR1(F2R) (purple) in the GSE15471, GSE62452, GSE28735 and GSE62165 datasets. For each set, expression in pancreatic cancer patients (dark) and non-tumor controls (light) is indicated. Student’s t test was used to determine the significance of the differences in expression between the tumor and non-tumor control groups. (**B**) Correlations between F2R and CD68 or CD163 expression (log2 scale) in the TCGA-PDAC, E-MTAB-6830 and GSE49149 datasets. On the lower right corner of each graph, *p*-values and Pearson correlation coefficients (R) are shown. (**C**) Pancreatic tumor microarray staining for CD68, CD163, PAR1, and H&E. Each layer was scanned separately and generated as virtual stacks in ImageJ. The virtual image stack on the right represents CD68 (green), CD163 (blue) and PAR1 (red) staining

To explore the functional interplay between PAR1, macrophages and tumor cells, we first determined the correlation between PAR1*/F2R* and macrophages in both tumor and control pancreatic tissues. To this end, four different PDAC gene expression sets were dichotomized for tissue status (i.e., tumor or non-tumor), after which the expression of the macrophage marker *CD68* and *F2R *(PAR1) was assessed. Both these markers were predominantly high in pancreatic tumor tissue (Fig. 1A). Next, we plotted the expression of the general macrophage marker CD68 and the tumor-associated macrophage marker CD163 versus *F2R* in tumor-only datasets (Fig. 1B). In these sets, CD68 and CD163 expression significantly correlated (Sup. Figure 1), and both markers correlated with PAR1*/F2R* expression (Fig. 1B). Subsequent immunohistochemical analysis of our in-house PDAC-TMA showed abundant PAR1, CD68 and CD163 expression, but no co-expression of PAR1 on either CD68- or CD163-positive cells (Fig. 1C). Overall, these data confirm that PAR1 is overexpressed in pancreatic tumor tissues and that PAR1 expression correlates with macrophage numbers in the tumor microenvironment, but that macrophages themselves are PAR1-negative.

### Macrophages induce EMT in a PAR1-dependent manner

**Fig. 2:**
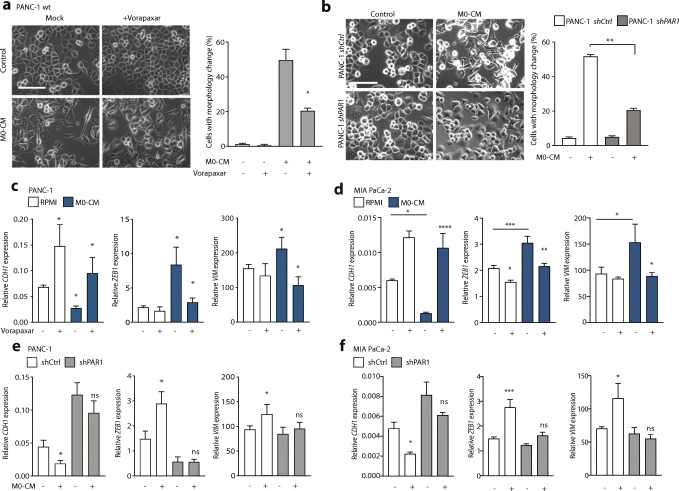
Macrophages induce EMT of pancreatic cancer cells in a PAR1-dependent manner. (**A**) Phase-contrast microscopic images of PANC-1 wildtype cells treated with control or M0-CM medium. PAR1 was inhibited by Vorapaxar (500 nM) using DMSO as a mock control. Spindle-shaped cells were quantified using the cell counter tool of Image J, after which the percentage per field was calculated according to the total number of cells. Shown is the mean ± SEM (*n* = 3); Student’s t test. (**B**) Morphology assessment of shCtrl and shPAR1 PANC-1 cells treated with M0-CM. Spindle-shaped cells were quantified using the cell counter tool of Image J, after which the percentage per field was calculated according to the total number of cells. Shown is the mean ± SEM (*n* = 3); Student’s t test. In panels A and B, magnification is 20x, and the scale bar indicates 50 μm. (**C-D**) Relative mRNA expression of CDH1, ZEB1 and VIM in RPMI-1640 (white) or M0-CM (blue) treated PANC-1 (**C**) and MIA PaCa-2 (**D**) cells. PAR1 was inhibited by Vorapaxar (500 nM) using DMSO as a mock control. Shown is the mean ± SEM (*n* = 4); Student’s t test. (**E-F**) Relative mRNA expression of CDH1, ZEB1 and VIM in RPMI-1640 (−) or M0-CM (+) treated PANC-1 (**E**) or MIA PaCa-2 (**F**) shCtrl (white) and shPAR1 (gray) cells. Shown is the mean ± SEM (*n* = 4); Student’s t test. Relative expression levels, as depicted in panels **C-F,** were calculated using the comparative threshold cycle (dCt method) and normalized to the expression of the reference gene TBP

To assess whether the observed correlation between PAR1 expression and macrophage infiltration has any functional consequence, we next focussed on the role of PAR1 in macrophage-tumor cell crosstalk. Conditioned medium (CM) from PMA-induced THP-1 monocytes (characterization shown in Sup. Figure 2A), hereafter called M0 macrophages, was applied to PANC-1 pancreatic cancer cells in the absence or presence of the PAR1 inhibitor Vorapaxar (Fig. 2A). We found that M0-CM induced fibroblast-like morphological changes in PANC-1 cells, which was reduced by Vorapaxar-dependent PAR1 inhibition (Fig. 2A). Importantly, the control conditioned medium (PANC-1-CM) did not induce any morphological changes (Sup. Figure 2B), excluding nutrient depletion to cause the effects of M0-CM. To confirm the specificity of the Vorapaxar results and to show that PAR1 acts on tumor cells, we next incubated PAR1-silenced PANC-1 cells (*shPAR1*; as generated previously [36]) and their controls (*shCtrl*) with M0-CM. Control cells showed similar morphological changes as non-transduced PANC-1 cells, whereas these changes were less apparent in *shPAR1* cells (Fig. 2B).

To molecularly characterize the macrophage-induced morphological changes, we measured the expression of cell state markers E-cadherin (*CDH1*), Zinc finger E-box binding homeobox 1 (*ZEB1*) and Vimentin (*VIM*). M0-CM treatment of PANC-1 and MIA PaCa-2 cells resulted in decreased *CDH1* expression and increased *ZEB1* and *VIM* expression (Fig. 2C-D). In line with the reduction in morphological changes observed by microscopy (Fig. 2A and B), PAR1 inhibition diminished M0-CM-induced changes of cell state markers. PAR1 silenced PANC-1 and MIA PaCa-2 cells showed similar changes as Vorapaxar treated control PAR1 expressing cells (Fig. 2E-F). M0-CM treatment decreased *CDH1* and increased *ZEB1* and *VIM* expression in *shCtrl* cells, but not in *shPAR1* cells (that already show enhanced epithelial characteristics as described previously [36]). Of note, and in line with our previous study on the contributions of PAR1 signaling to tumor cell state [36], we found that also in the absence of M0-CM inhibition of PAR1 (using Vorapaxar) resulted in an epithelial phenotype shift in cancer cells.

**Fig. 3:**
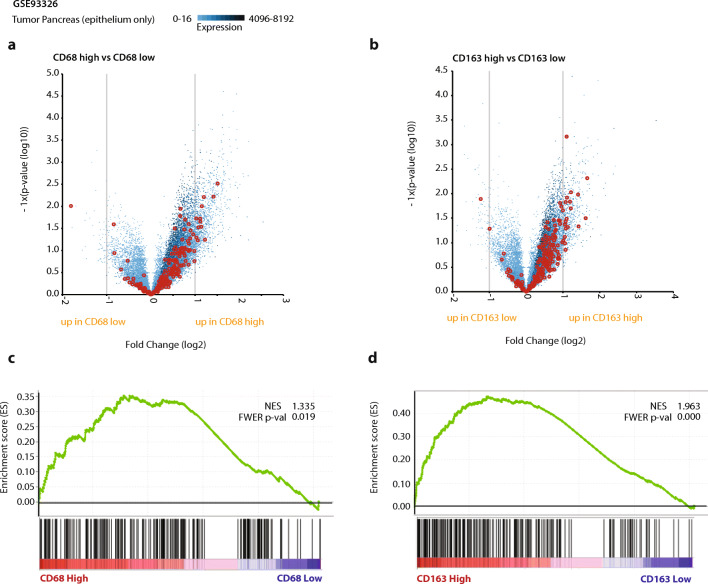
Macrophage influx correlates with EMT of cancer cells. High CD68 and CD163 macrophage marker expression in stroma indicate enhanced EMT in epithelial cells. (**A-B**) Differential expression analysis of tumor cells dichotomized on the stromal expression of CD68 (**A**) or CD163 (**B**) with genes from the Hallmark_EMT signature highlighted in red. (**C-D**) Gene Set Enrichment Analysis (GSEA) results for human PDAC cells from the GSE93326 expression set (dichotomized for median CD68 (**C**) or CD163 (**D**)) with the Hallmark_EMT signature. Normalized Enhancement Score (NES) and Family-Wise Error Rate (FWER) *p*-values are shown on the enrichment plots

To substantiate our findings that loss of PAR1 seems to block a macrophage-mediated mesenchymal cell state transition, we correlated stromal macrophage content with tumor cell phenotype in PDAC. To avoid confounding by stromal cells expressing mesenchymal markers, we performed these analyses using a laser capture micro-dissected gene expression set (GSE93326) containing paired stromal and tumor epithelium samples. We used the median expression of *CD68* and *CD163* from the stromal samples to dichotomize the matched tumor samples. Next, we generated differential gene expression plots between CD68 (Fig. 3A) or CD163 (Fig. 3B) high and low tumor samples. The association with EMT was highlighted using the Hallmark_EMT gene set (derived from MSigDB). Interestingly, the expression of most EMT related genes was increased in the CD68 or CD163 high groups. In line with these findings, gene set enrichment analysis (GSEA) showed that both CD68 and CD163 expression positively correlated with epithelial signatures (Fig. 3C and D). Overall, these data suggest that macrophage influx correlates with a mesenchymal tumor state.

### Macrophage-secreted MMP9 activates PAR1 and drives EMT

**Fig. 4:**
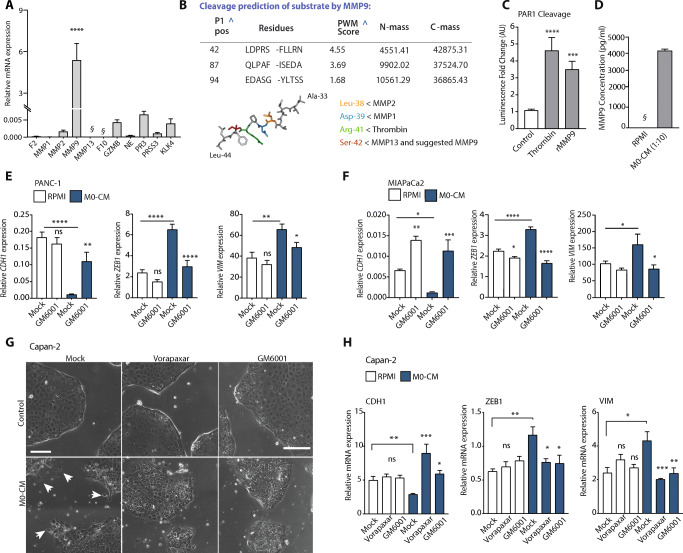
Macrophage-secreted MMP9 activates PAR1. (**A**) Relative mRNA expression levels of PAR1-cleaving proteases in M0 macrophages. The expression levels of F2 (Thrombin), MMP1, MMP2, MMP9, MMP13, F10 (FX), GZMB, NE, PR3, PRSS3 and KLK4 are shown as mean ± SEM (*n* = 4); Student’s t test. § indicates signals below the detection limit. (**B**) MMP9 cleavage prediction of the PAR1 N-terminal amino acid sequence, derived from the FASTA sequence of Uniprot ID: P25116. P1 position, sequence, and PWM (position weight matrices) are shown together with the mass of the N- and C-terminal sequences after cleavage. Below the table, a stick representation for the PAR1 N-terminal amino acid sequence, where protease cleavage sites are concentrated, is shown. In this representation, locations for MMP2 (yellow), MMP1 (blue), Thrombin (Green) and MMP13, together with MMP9 (red) are indicated. (**C**) Quantification of PAR1 cleavage with 100 nM recombinant MMP9 (rMMP9) and 0.1 U/ml Thrombin in PAR1-SEAP assays. *N* = 4. Error bars show mean ± SEM. One-way ANOVA. (**D**) MMP9 levels in M0-CM and RPMI-1640 media. Shown is the mean ± SEM (*n* = 4); Student’s t test. § indicates signals below the detection limit. (**E-F**) Relative mRNA expression of CDH1, ZEB1 and VIM in RPMI-1640 (white) or M0-CM (blue) treated PANC-1 (**E**) and MIA PaCa-2 (**F**) cells. MMP9 was inhibited by GM6001 (5 μM) using DMSO as a mock control. Shown is the mean ± SEM (*n* = 4); Student’s t test. (**G**) Phase-contrast microscope image of Capan-2 cells after control (1:1 DMEM+RPMI-1640) and M0-CM treatment. PAR1 was inhibited by Vorapaxar (500 nM), MMP9 was inhibited by GM6001 (5 μM), and DMSO served as a mock control. Shown are images at t = 72 h after the addition of M0-CM. Magnification is 10x, and scale bars indicate 100 μm. (**H**) Relative mRNA expression of CDH1, ZEB1 and VIM in RPMI-1640 (white) or M0-CM (blue) treated Capan-2 cells. PAR1 was inhibited by Vorapaxar (500 nM), MMP9 was inhibited by GM6001 (5 μM), and DMSO served as a mock control. Shown is the mean ± SEM (*n* = 4); Student’s t test. Relative expression levels, as depicted in panels **E**, **F,** and **H,** were calculated using the comparative threshold cycle (dCt method) and normalized to the expression of the reference gene TBP

In order to identify the PAR1 agonist that mediates cancer cell differentiation, we next analyzed expression levels of all confirmed or suggested PAR1-cleaving proteases in M0 macrophages. Although several proteases were found to be expressed by M0 macrophages, MMP9 was most abundantly expressed with levels approximately 1000-fold higher than those of granzyme B, proteinase 3 and kallikrein 4 (Fig. 4A). Although MMP9 has been suggested to act as a PAR1 cleaving protease [37, 38], detailed experimental proof for its activity on PAR1 has so far not been provided. Therefore, we next assessed whether the N-terminal tethered ligand of PAR1 contains a putative MMP9 cleavage site using the CleavPredict algorithm [27]. As shown in Fig. 4B, this in silico analysis identified three potential MMP9 cleavage sites in the N-terminal part of PAR1 with the most robust proteolytic cleavage for the P1 position at Serine 42. Interestingly, this predicted MMP9 cleavage site lies directly adjacent to the thrombin cleavage site and is similar to the proposed MMP13 cleavage site [39]. To demonstrate that MMP9 can cleave PAR1, SEAP-PAR1 reporter cells [35] were incubated with recombinant MMP9, thrombin (positive control), or PBS (negative control). Both recombinant MMP9 and thrombin efficiently induced the proteolytic release of SEAP, indicative of PAR1 cleavage (Fig. 4C). To confirm that macrophage-secreted MMP9 is indeed responsible for the observed macrophage-dependent mesenchymal transition of PANC-1 and MIA PaCa-2 cells, we next determined MMP9 levels in M0-CM and found that the medium indeed contains high MMP9 levels (Fig. 4D). Finally, we assessed whether MMP9 inhibition could block the M0-CM-induced mesenchymal transition. As shown in Fig. 4E and F, the effects of MMP9 inhibition with GM6001 indeed mimic that of Vorapaxar and significantly limit the M0-CM-induced decrease in *CDH1* and increases in *ZEB1* and *VIM* expression. As for Fig. 2 and our previous work [36], inhibition of MMP9 in the absence of any macrophage-derived cues conferred an increased epithelial phenotype in MIA PaCa-2 cells (Fig. 4F).

To confirm the role of the MMP9-PAR1 axis in mesenchymal transition and to assess the general applicability of our findings, we next used Capan-2 cells that are particularly epithelial and could, therefore, be expected to be more resilient to full transition into a mesenchymal state [40]. Capan-2 cells were incubated in M0-CM or control medium. M0-CM indeed induced morphological changes (Fig. 4G, white arrows), and these changes were accompanied by decreased *CDH1* and increased *ZEB1* and Vimentin (*VIM*) expression (Fig. 4H). Vorapaxar and GM6001 treatment significantly decreased the M0-CM-induced morphological changes (Sup. Figure 3A). Similar to PANC-1 and MIA PaCa-2 cells, direct PAR1 inhibition by Vorapaxar or indirect inhibition by GM6001 prevented M0-CM-induced mesenchymal transition of Capan-2 cells as evident from reduced morphological changes as well as reduced expression of the cell state markers *CDH1*, *ZEB1* and *VIM*. Considering these results, a picture emerges that macrophage-secreted MMP9 activates PAR1, which further orchestrates mesenchymal differentiation.

### PAR1 signaling limits macrophage-induced cytotoxicity

**Fig. 5:**
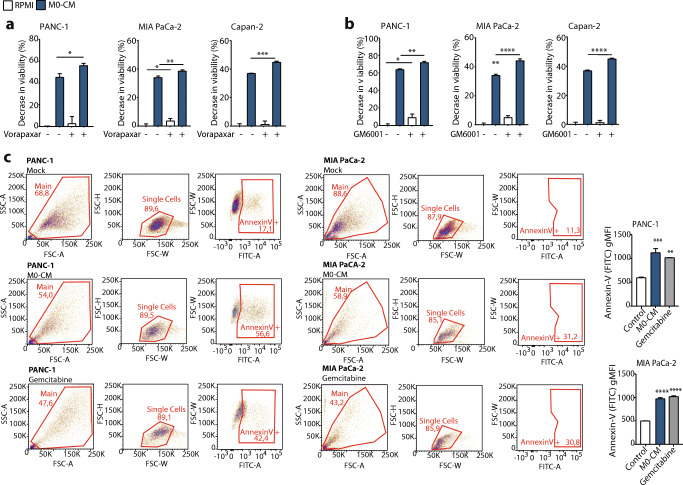
The PAR1-MMP9 axis reduces macrophage induced cytotoxicity. (**A-B**) MTT viability assay of PANC-1, MIA PaCa-2 and Capan-2 cells in RPMI-1640 (white) or M0-CM (blue) medium. Shown in the effect of PAR1 inhibition with 500 nM Vorapaxar (**A**) or MMP9 inhibition with 5 μM GM6001 (**B**). Decreased viability is calculated relative to the viability of control-treated cells. Shown is the mean ± SEM (*n* = 6); One-way ANOVA. (**C**) Annexin V-FITC+ cells in mock (RPMI-1640), M0-CM, or positive-control (Gemcitabine) treated PANC-1 and MIA PaCa-2 cells at t = 48 h. Left panel: gating strategy for the Annexin V+ population. The FITC gate was set on antibody controls. Right panel: geometric Mean Fluorescent Intensity (gMFI) on the FITC channel (Annexin V density) for Mock, M0-CM, and positive-control treated cells from the previous panel is given. Shown is the mean ± SEM (*n* = 3); One-way ANOVA

Mesenchymal transition is known to cause drug resistance [41], and we aimed to assess whether macrophage-MMP9-PAR1 driven EMT also impacts resistance. Contrary to our expectations, however, we found that M0-CM already caused a substantial decrease in cell viability by itself (Fig. 5A and Sup. Figure 3B-C). The M0-CM induced cytotoxicity was dependent on the MMP9-PAR1 signaling axis as both Vorapaxar and GM6001 significantly potentiated macrophage-induced cytotoxicity in PANC-1, MIA PaCa-2 and Capan-2 cells (Fig. 5A-B, and Sup. Figure 3B-C). To exclude that the decreased viability after M0-CM medium transfer may be due to nutrient depletion, we compared time-matched medium form PANC-1 cells (PANC-1-CM) with M0-CM and found that the decrease in viability was specific to M0-CM (Sup. Figure 4). To formally discriminate between cytotoxicity or decreased proliferation after M0-CM treatment, we analyzed Annexin-V positivity in control, and M0-CM-treated PANC-1 and MIA PaCa-2 cells. M0-CM treatment indeed induced Annexin-V positivity in both cell lines, showing that M0-CM drives PDAC cells into apoptosis (Fig. 5C). To substantiate these findings, we next analyzed the effect of PAR1 silencing on M0-CM-induced cytotoxicity. Under M0-CM treatment, the addition of GM6001 only increased cytotoxicity in *shCtrl* cells but did not change the response in *shPAR1* PANC-1, MIA PaCa-2 and Capan-2 cells (Sup. Figure 5A-B). Overall, these data suggest that the MMP9-PAR1 axis allows tumor cells to escape macrophage-dependent cell death.

### Mesenchymal transition is a prerequisite for tumor cells to survive macrophage-induced cytotoxicity

**Fig. 6:**
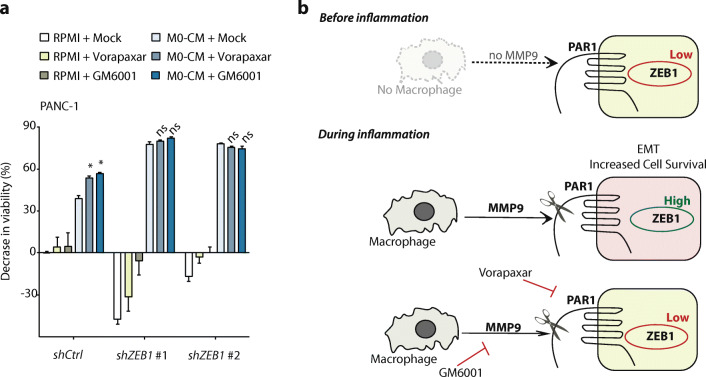
Mesenchymal transition protects tumor cells from macrophage induced cytotoxicity. (**A**) MTT viability assays of PANC-1 shCtrl, shZEB1 #1 and shZEB1 #2 cells treated with RPMI-1640 (green bars) or M0-CM (blue bars). PAR1 was inhibited by Vorapaxar (500 nM), MMP9 was inhibited by GM6001 (5 μM), and DMSO served as a mock control. Decreased viability was calculated relative to the viability of control-treated cells. Shown is the mean ± SEM (*n* = 6); One-way ANOVA. (**B**) Schematic representation showing that macrophages secrete MMP9 that activates PAR1 on pancreatic cancer cells, thereby inducing mesenchymal transition and subsequent resistance to macrophage-induced cytotoxicity

Activation of PAR1 by macrophage-secreted MMP9 results in increased mesenchymal transition and reduced PDAC cell death. To functionally ascertain that the mesenchymal transition is causal in the escape of PDAC cells from macrophage-dependent cell death, we generated *ZEB1*-silenced PANC-1 cells to block their capacity to undergo EMT and, subsequently, exposed these cells to M0-CM. *ZEB1*-silenced cells (knockdown efficiency depicted in Sup. Figure 6) indeed showed largely diminished cell viability in response to M0-CM compared to control silenced cells that are capable of mesenchymal transition (Fig. 6A). Moreover, targeting the MMP9-PAR1 axis in ZEB1-silenced cells did not further affect cell viability, confirming that MMP9-PAR1-dependent mesenchymal transition is the mechanism by which tumor cells escape macrophage-dependent cytotoxicity (Fig. 6A). Overall, these data thus show that mesenchymal transition protects tumor cells from macrophage-induced cytotoxicity (Fig. 6B).

## Discussion

Previous work has shown that stromal PAR1 ablation limits pancreatic cancer progression and potentiates gemcitabine efficacy [23]. In these experiments, diminished tumor growth was accompanied by reduced macrophage infiltration into the tumor [23], and macrophages were found to play a key role in PDAC development and progression [42]. Consequently, we hypothesized that PAR1-dependent macrophage-tumor cell crosstalk may contribute to the poor prognosis of PDAC. Using patient tumor biopsies, we confirmed that PAR1 is overexpressed in pancreatic tumor tissues and that its expression correlates with macrophage infiltration into the tumor microenvironment. Of note, we found that macrophage-derived signals act on PAR1 to mediate tumor-promoting effects. This is of particular interest in light of the dichotomous contributions of macrophages to cancer.

Early during tumor development, bone marrow-derived monocytes are recruited into the tumor microenvironment as an anti-tumor immune response. These cells then differentiate into naive (M0) macrophages, and depending on specific signals from the tumor microenvironment, they subsequently polarize into M2 or tumor-associated macrophages (TAMs), which have been described to induce EMT in cancer cells and to drive metastasis and drug resistance [15–17]. Here, we assessed the functional interplay between M0 macrophages, PAR1 and tumor cells and found that M0 macrophages induce distinct morphological changes in PDAC cells reminiscent of EMT. This effect was dependent on an MMP9-PAR1 signaling axis, and subsequent experiments with ZEB1-silenced PDAC cells further underscored the contributions of mesenchymal transition programs to the escape of tumor cells from macrophage-induced cytotoxicity. This suggests that early in the sequence of macrophage recruitment to the tumor microenvironment, M0 macrophage differentiation results in MMP9-PAR1-EMT dependent crosstalk that facilitates tumor progression.

We found that M0 macrophages secrete a PAR1 agonist leading to PAR1-dependent mesenchymal transition of pancreatic cancer cells. Although thrombin is the first described and best-known PAR-1 agonist, more recently, other agonists like matrix metalloproteases (MMPs) and kallikreins have been described [22]. Of these potential PAR1 agonists, we here identified MMP9 as the most likely endogenous agonist secreted by M0 macrophages. Based on MMP9-dependent PAR1 internalization, it has previously been hypothesized that MMP9 may activate PAR1 [38], but functional evidence was not provided. Here, we substantiate these findings by showing that MMP9 indeed cleaves PAR1. Our in silico analysis identified Ser-42 of PAR1 as the most likely MMP9 cleavage site, which is particularly interesting as Ser-42 was previously identified as an MMP13 cleavage site leading to pathological activation of Gαq- and ErbB receptor-dependent pathways in the heart [39], suggesting that MMP9-dependent cleavage indeed activates PAR1. In line with this notion, we found that MMP9 inhibition mimics PAR1 inhibition. Indeed, GM6001 and Vorapaxar both block M0-CM-induced mesenchymal transition and prevent macrophage-induced cytotoxicity. It should be noted that the genetic silencing of PAR1 by shRNA transduction and pharmacological inhibition of PAR1 (Vorapaxar) or MMP9 (GM6001) did not completely reverse M0-CM-induced EMT. Most likely, this may be due to residual PAR1 expression/signaling, although it cannot be ruled out that macrophages secrete alternative mediators that induce morphological changes independent of PAR1.

In the current study, we revealed a tumor-promoting effect of the MMP9/PAR1 axis, suggesting that targeting this axis may have clinical benefit. Although PAR1 is generally considered to promote cancer progression [43] PAR1 may, however, not be the most attractive target to pursue in a cancer setting due to recent observations showing that genetic elimination of PAR1, in fact, aggravates tumor development [36, 44]. The context-dependent role of PAR1 in tumor biology is not fully understood, but it is well conceivable that the outcome of PAR1 activation depends on the activating agonists. Indeed, PAR1 is well known to exert biased agonism, a process in which different agonists activate multiple signaling pathways that have distinct or even opposite effects on cell function [35]. Instead of PAR1, it may thus be better to target MMP9. Interestingly, MMP9 levels have already been shown to correlate with lymph node involvement and the occurrence of distant metastases in pancreatic cancer patients [45]. Moreover, tumor cell MMP9 levels [46], as well as preoperative serum MMP9 concentrations [47], have been found to significantly correlate with the survival of pancreatic cancer patients, identifying MMP9 as a prognostic marker for PDAC survival. Although the clinical efficacy of MMP9 inhibition in PDAC remains to be established, recent observations that selective MMP9 inhibition in combination with mFOLFOX6 showed encouraging clinical activity without additional toxicity in patients with HER2-negative gastric and gastric/gastroesophageal junction adenocarcinomas [48], suggest that MMP9 inhibition may be promising for enhancing combined therapeutic benefits.

## Conclusion

In the early stages of tumor progression, macrophages exert anti-tumor effects. Here we show that in response to macrophage-secreted MMP9, tumor cells undergo mesenchymal transition in a PAR1-dependent manner. This adds to our understanding of the pro-tumor contributions of macrophages and may explain the contradictory contributions of macrophages to pancreatic as well as other cancers.

## Electronic supplementary material

ESM 1 Correlation of CD68 with CD163 expression (on log2 scale) in the TCGA-PDAC, E-MTAB-6830, and GSE49149 datasets. On the lower right corner of each graph, *p*-values and Pearson correlation coefficients (R) are shown. (PNG 51 kb)

High Resolution Image (EPS 1709 kb)

ESM 2 (**A**) Relative mRNA expression of CD68, CD163, and CD206 in M0 and M2 macrophages. Shown is the mean ± SEM (*n* = 4); One-way ANOVA. Relative expression levels, as depicted in this panel, were calculated using the comparative threshold cycle (dCt method) and were normalized for expression of the reference gene TBP. (**B**) Phase-contrast microscope images of PANC-1 cells under RPMI and 1:1 PANC-CM treatment. Images are taken at t = 72 h. Magnification is at 10X, and scale bars indicate 100 μm. (PNG 836 kb)

High Resolution Image (EPS 4768 kb)

ESM 3 (**A**) Quantification of morphological changes of Capan-2 cells treated with RPMI (white) or M0-CM (blue). PAR1 was inhibited by Vorapaxar (500 nM), MMP9 was inhibited by GM6001 (5 μM), and DMSO served as a mock control. Quantification is done at t = 72 h (images are shown in Fig. 3G). Shown is the mean ± SEM (n = 4); One-way ANOVA. (**B**) Cell numbers of Capan-2 cells after RPMI (white) or M0-CM (blue) treatment calculated based on the difference in total cell area at t = 96 versus t = 0 H*. par*1 was inhibited by Vorapaxar (500 nM), MMP9 was inhibited by GM6001 (5 μM), and DMSO served as a mock control. Shown is the mean ± SEM (*n* = 3); Student’s t test. (**C**) Crystal Violet assays of PANC-1, MIA PaCa-2, and Capan-2 cells treated with RPMI (white) or M0-CM (blue). Shown is the effect of PAR1 inhibition with 500 nM Vorapaxar (**A**) or MMP9 inhibition with 5 μM GM6001. (PNG 50 kb)

High Resolution Image (EPS 1823 kb)

ESM 4 MTT viability assays of PANC-1 cells treated with RPMI, PANC-CM, and M0-CM. Shown is the mean ± SEM (n = 4); One-way ANOVA. (PNG 11 kb)

High Resolution Image (EPS 1323 kb)

ESM 5 MTT **(A)** and Crystal Violet (**B**) cell viability assays of PANC-1, MIA PaCa-2, and Capan-2 cells treated with RPMI (white) or M0-CM (blue). PAR1 was inhibited by Vorapaxar (500 nM), MMP9 was inhibited by GM6001 (5 μM), and DMSO served as a mock control. Shown is the mean ± SEM (*n* = 6); One-way ANOVA. (PNG 55 kb)

High Resolution Image (EPS 1868 kb)

ESM 6 Relative ZEB1 mRNA expression of control (shCtrl) and ZEB1 (shZEB1 #1 and shZEB1 #2) silenced PANC-1 cells. Shown is the mean ± SEM (n = 4); One-way ANOVA. Relative expression level, as depicted in this panel, was calculated using the comparative threshold cycle (dCt method) and was normalized for expression of the reference gene TBP. (PNG 11 kb)

High Resolution Image (EPS 1311 kb)
