# Supplementary figures and images for: Macrophage-secreted MMP9 induces mesenchymal transition in pancreatic cancer cells via PAR1 activation
Source: Cell Oncol (Dordr). 2020 Aug 18;43(6):1161–74. doi: 10.1007/s13402-020-00549-x (PMC7717035; doi:10.1007/s13402-020-00549-x)

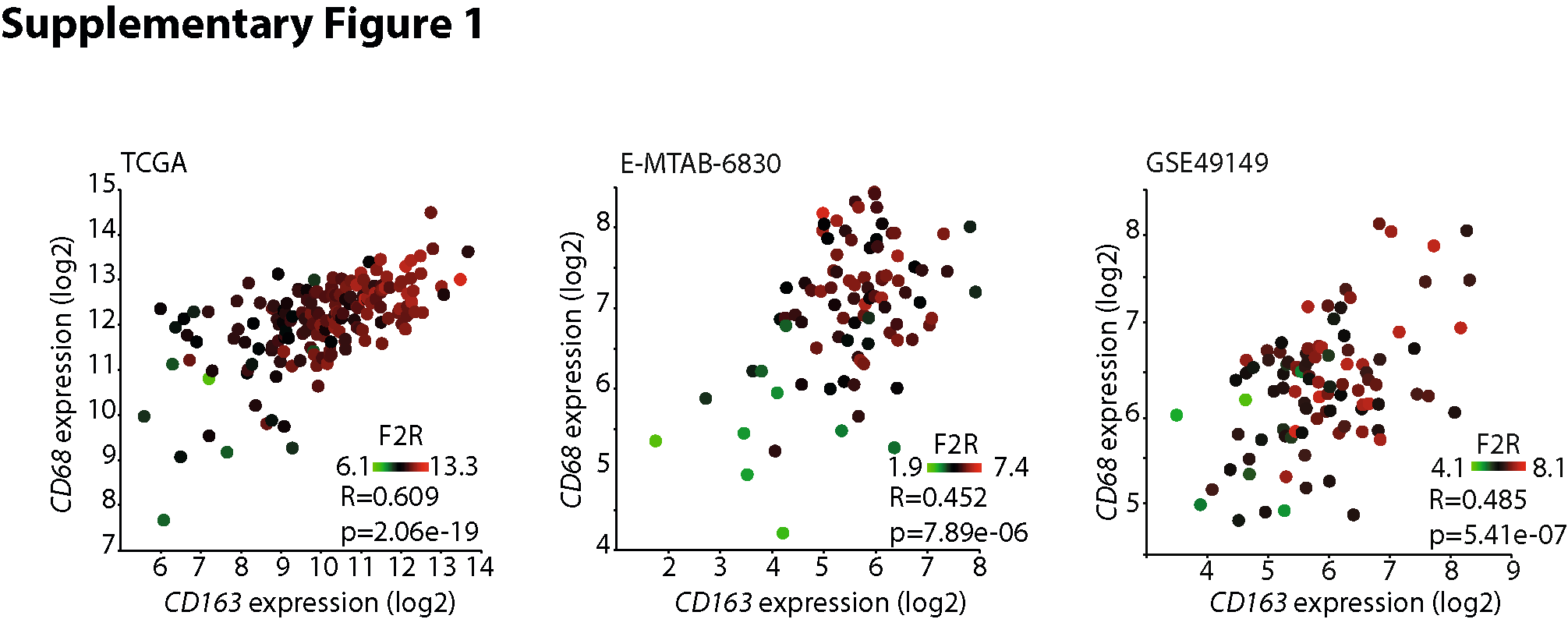

Supplement: Supplementary file 1 — Correlation of CD68 with CD163 expression (on log2 scale) in the TCGA-PDAC, E-MTAB-6830, and GSE49149 datasets. On the lower right corner of each graph, p-values and Pearson correlation coefficients (R) are shown. (PNG 51 kb) [file 13402_2020_549_Fig7_ESM.png]

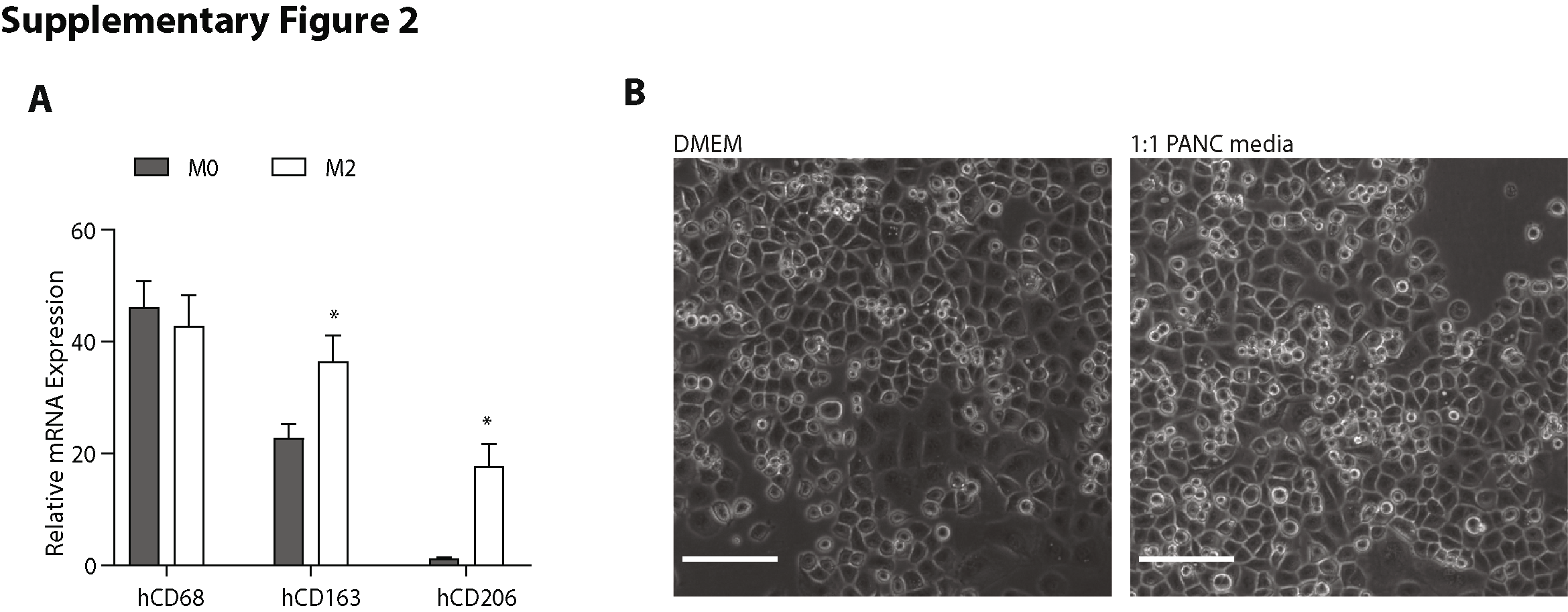

Supplement: Supplementary file 3 — (A) Relative mRNA expression of CD68, CD163, and CD206 in M0 and M2 macrophages. Shown is the mean ± SEM (n = 4); One-way ANOVA. Relative expression levels, as depicted in this panel, were calculated using the comparative threshold cycle (dCt method) and were normalized for expression of the reference gene TBP. (B) Phase-contrast microscope images of PANC-1 cells under RPMI and 1:1 PANC-CM treatment. Images are taken at t = 72 h. Magnification is at 10X, and scale bars indicate 100 μm. (PNG 836 kb) [file 13402_2020_549_Fig8_ESM.png]

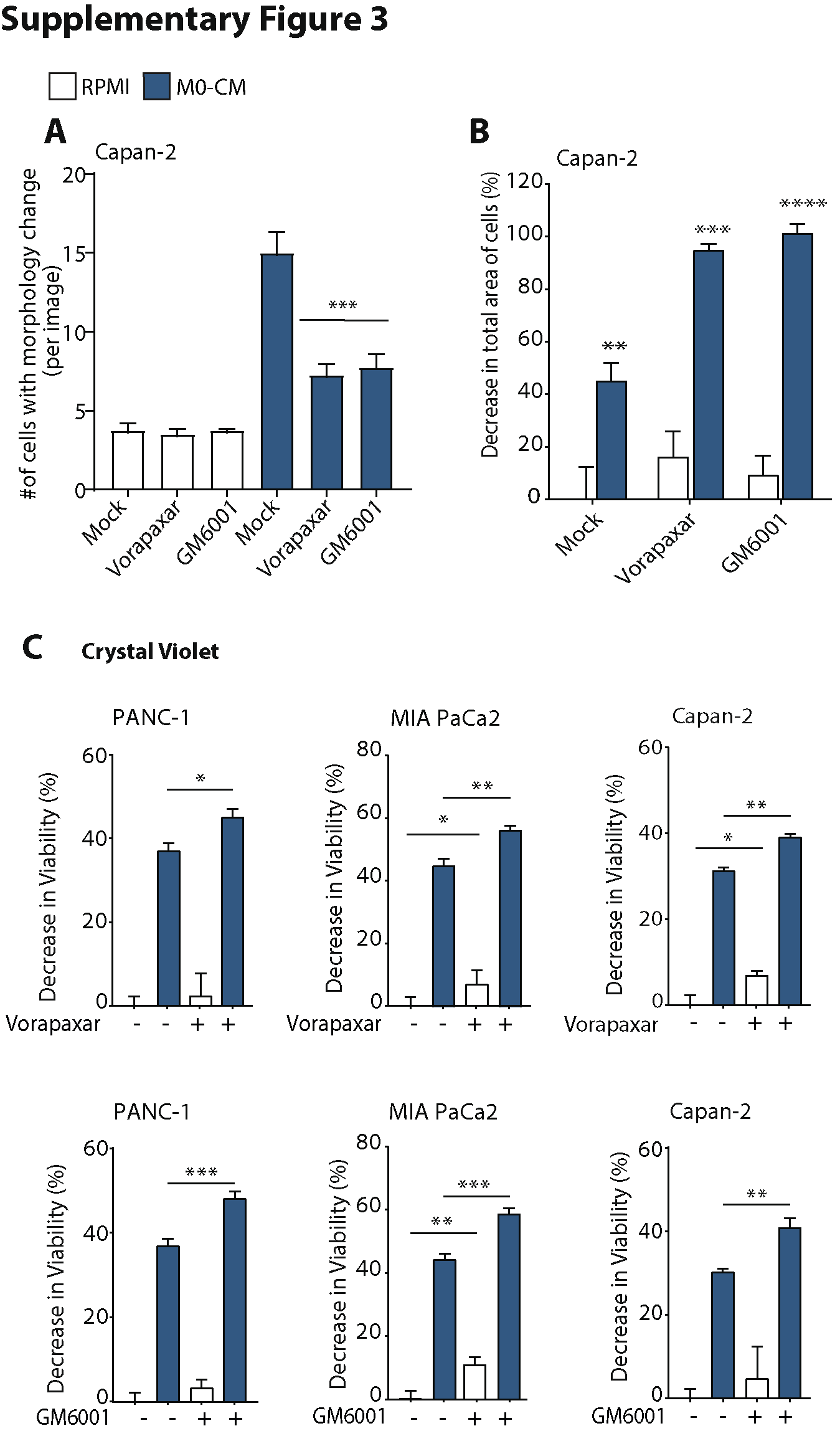

Supplement: Supplementary file 5 — (A) Quantification of morphological changes of Capan-2 cells treated with RPMI (white) or M0-CM (blue). PAR1 was inhibited by Vorapaxar (500 nM), MMP9 was inhibited by GM6001 (5 μM), and DMSO served as a mock control. Quantification is done at t = 72 h (images are shown in Fig. 3G). Shown is the mean ± SEM (n = 4); One-way ANOVA. (B) Cell numbers of Capan-2 cells after RPMI (white) or M0-CM (blue) treatment calculated based on the difference in total cell area at t = 96 versus t = 0 H. par1 was inhibited by Vorapaxar (500 nM), MMP9 was inhibited by GM6001 (5 μM), and DMSO served as a mock control. Shown is the mean ± SEM (n = 3); Student’s t test. (C) Crystal Violet assays of PANC-1, MIA PaCa-2, and Capan-2 cells treated with RPMI (white) or M0-CM (blue). Shown is the effect of PAR1 inhibition with 500 nM Vorapaxar (A) or MMP9 inhibition with 5 μM GM6001. (PNG 50 kb) [file 13402_2020_549_Fig9_ESM.png]

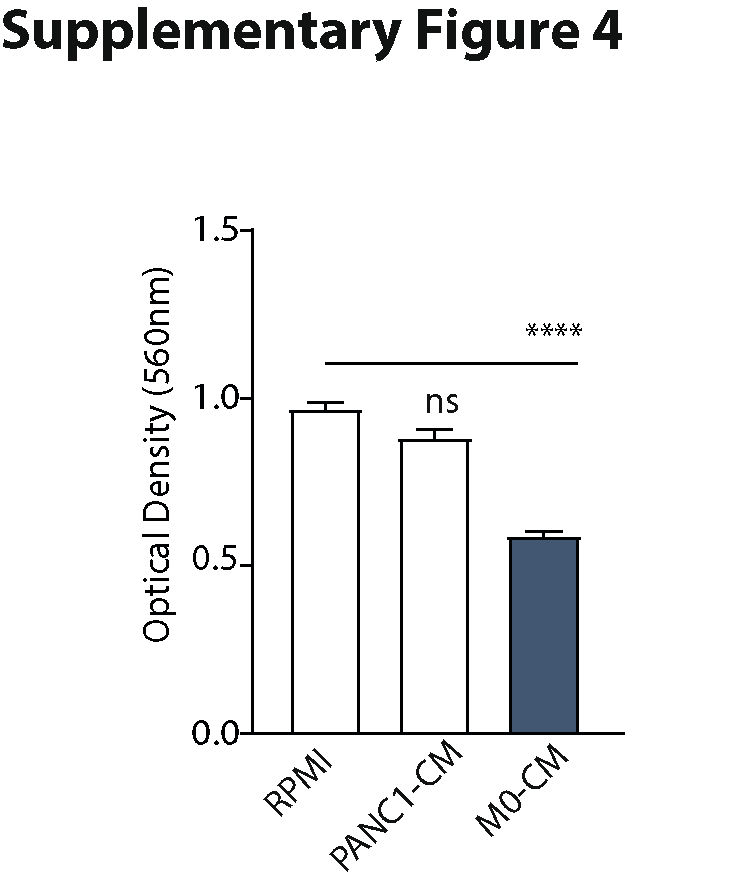

Supplement: Supplementary file 7 — MTT viability assays of PANC-1 cells treated with RPMI, PANC-CM, and M0-CM. Shown is the mean ± SEM (n = 4); One-way ANOVA. (PNG 11 kb) [file 13402_2020_549_Fig10_ESM.png]

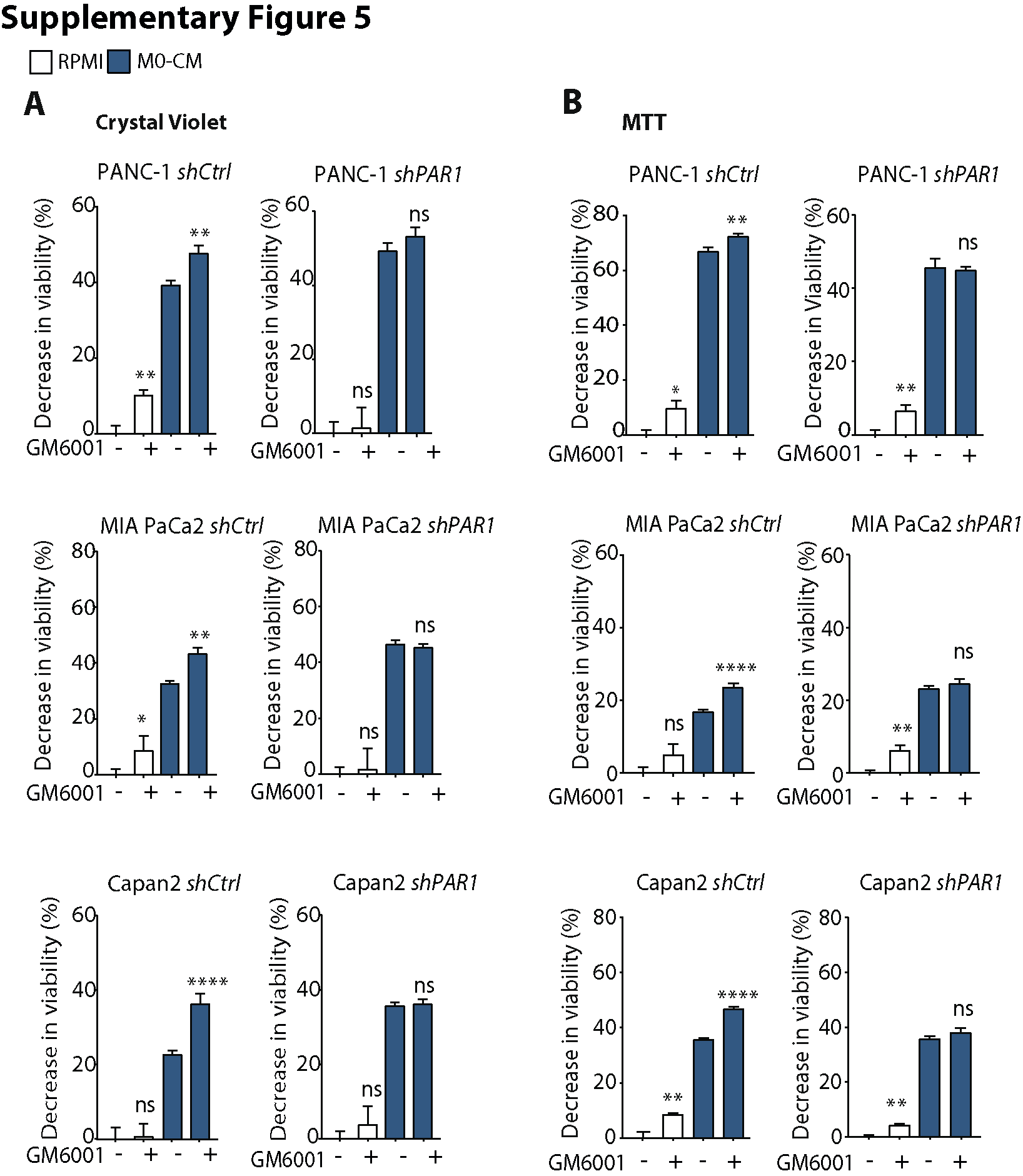

Supplement: Supplementary file 9 — MTT (A) and Crystal Violet (B) cell viability assays of PANC-1, MIA PaCa-2, and Capan-2 cells treated with RPMI (white) or M0-CM (blue). PAR1 was inhibited by Vorapaxar (500 nM), MMP9 was inhibited by GM6001 (5 μM), and DMSO served as a mock control. Shown is the mean ± SEM (n = 6); One-way ANOVA. (PNG 55 kb) [file 13402_2020_549_Fig11_ESM.png]

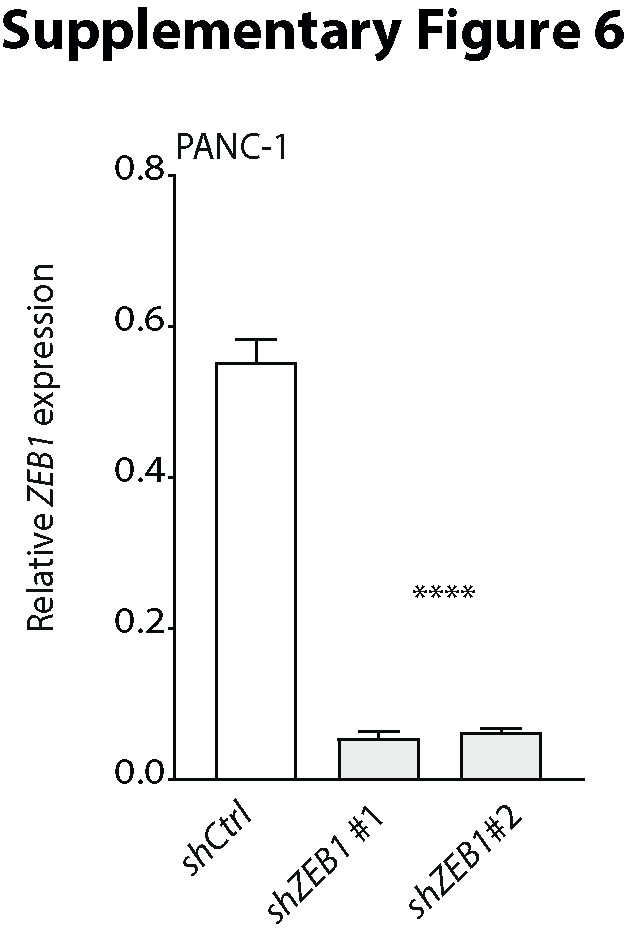

Supplement: Supplementary file 11 — Relative ZEB1 mRNA expression of control (shCtrl) and ZEB1 (shZEB1 #1 and shZEB1 #2) silenced PANC-1 cells. Shown is the mean ± SEM (n = 4); One-way ANOVA. Relative expression level, as depicted in this panel, was calculated using the comparative threshold cycle (dCt method) and was normalized for expression of the reference gene TBP. (PNG 11 kb) [file 13402_2020_549_Fig12_ESM.png]
